# Comparative Performance of Modified Kenneth Jones Criteria Scoring, World Health Organization Criteria, and Antibodies in Lymphocyte Supernatant for Diagnosing Tuberculosis in Severely Malnourished Children Presenting With Pneumonia

**DOI:** 10.3389/fped.2019.00406

**Published:** 2019-10-01

**Authors:** Mohammod Jobayer Chisti, Abu S. M. S. B. Shahid, K. M. Shahunja, Sayera Banu, Rubhana Raqib, Lubaba Shahrin, Shoeb Bin Islam, Sharifuzzaman, Haimanti Saha, Tahmina Alam, Muhammad Waliur Rahman, Sharika Nuzhat, Farzana Afroze, Monira Sarmin, Tahmeed Ahmed

**Affiliations:** International Centre for Diarrhoeal Disease Research, Bangladesh (icddr,b), Dhaka, Bangladesh

**Keywords:** antibodies in lymphocyte supernatant, modified Kenneth Jones criteria scoring, severely malnourished children, tuberculosis, World Health Organization criteria

## Abstract

**Background:** The diagnosis of childhood tuberculosis (TB) can be difficult in severely malnourished children. This is mainly due to the fact of our perceived notion that clinical signs of TB are often subtle in severely malnourished children and we may rely on laboratory investigation for the diagnosis. However, comparative data on the performance of clinical and laboratory diagnostics of TB in such population are also very limited.

**Objectives:** To compare the performance of composite clinical criteria and a technique that measures antibodies in lymphocyte supernatant (ALS) for the diagnosis of TB in severely malnourished children with pneumonia.

**Methods:** Severely malnourished children under five with radiological pneumonia admitted to the Dhaka Hospital of International Centre for Diarrhoeal Disease Research, Bangladesh were enrolled consecutively following informed consent. We collected venous blood for ALS, gastric lavage fluid and induced sputum for microscopy, mycobacterial culture, and real-time PCR by Xpert MTB/RIF. We compared the sensitivity, specificity, positive, and negative predictive values, and accuracy of modified Kenneth Jones criteria (MKJC) score, World Health Organization (WHO) criteria, and ALS in diagnosing TB in severely malnourished children with pneumonia for “Confirmed TB” and “All TB” (“Confirmed TB” plus “Probable TB”) vs. “Not TB.”

**Results:** Compared to culture confirmed TB, the sensitivity, and specificity (95% CI) for MKJC were 60 (27–86) and 84 (79–87)% and for WHO criteria were 40 (14–73) and 84 (80–87)%, respectively. Compared to culture and/or Xpert MTB/RIF positive TB, the sensitivity and specificity (95% CI) for the criteria were 37 (20–58) and 84 (79–87)%; and 22 (9–43) and 83 (79–87)%, respectively. For both these comparisons, the sensitivity and specificity of ALS were 50 (14–86) and 60 (53–67)%, respectively.

**Conclusion:** Our data suggest that WHO criteria and MKJC scoring mainly based on clinical criteria are more useful than ALS in diagnosing TB in young severely malnourished children with pneumonia. The results underscore the importance of using clinical criteria for the diagnosis of TB in severely malnourished children that may help to minimize the chance of over treatment with anti-TB in such population, especially in resource limited TB endemic settings.

## Introduction

Although, childhood tuberculosis (TB) is associated with high deaths in TB endemic settings (1), early diagnosis of TB in children is really intriguing especially on the basis of laboratory evidence. In developing countries, clinicians often rely on a combination of epidemiology, medical history including history of exposure, clinical features, chest x-ray findings, and tuberculin skin test (TST) in making a diagnosis and thereby treating of childhood TB (2). Sputum microscopy is routinely used in the diagnosis of adult TB; however, collection of sputum for microscopy is difficult in children who may not produce reliable samples. Even when collection of sample (gastric lavage/induced sputum) is possible, microscopy often comes with negative results due to pauci-bacillary nature of childhood TB. The conventional culture for the isolation of *Mycobacterium tuberculosis* takes as long as 8–12 weeks. Recent wonder of TB diagnostics, real-time PCR by Xpert MTB/RIF which only takes 2 h to provide results, is expensive and has poorer sensitivity in children (3) compared to adults (4). However, in both the diagnostics, collection of high quality sample is imperative. Sample from sick malnourished children requires at least 3–4 h fasting with careful monitoring. In this contexts, modified Kenneth Jones criteria (MKJC) score (5), and World Health Organization (WHO) criteria (6) for childhood TB diagnosis, both of which mainly based on simple clinical data to determine the likelihood that a child has tuberculosis in conjunction with response to therapy and nutritional status (7), might have greater value in settings where microscopy is negative or Xpert MTB/RIF is out of reach. Recently, antibodies in lymphocyte supernatant (ALS) has been reported to correlate with clinical diagnoses of TB in adults (8, 9) and children (10), but it did not perform well when it was compared with microbiologically confirmed childhood TB in severely malnourished children (11). In this background, the aim of this analysis was to evaluate the relative performance of MKJC score, WHO criteria, and ALS in the diagnosis of childhood TB in comparison with culture and Xpert MTB/RIF.

## Materials and Methods

### Ethics Statement

The study (protocol number: PR-10067) was approved by the Research Review Committee (RRC) and the Ethical Review Committee (ERC) of icddr,b. Written informed consent was obtained from parents or guardians of each of the participating children; children whose caregivers did not give consent were not enrolled.

### Study Design

The information of study population, study settings, study sample, and patient management has been described in a recently published study (11). The sample for ALS was taken from blood of the study population in addition to gastric lavage fluid and induced sputum for microscopy, mycobacterial culture, and real-time PCR by Xpert MTB/RIF. Using culture and/or Xpert MTB/RIF positivity as the reference, we compared the sensitivity, specificity, positive, and negative predictive values, and accuracy of modified Kenneth Jones criteria (MKJC) (5), and World Health Organization (WHO) criteria (6), and ALS for the diagnosis of TB in severely malnourished children presenting with pneumonia. Laboratory procedure for ALS has been described earlier by Raqib et al. (10). Briefly, for ALS assay 3.0 ml blood was taken from the patient with adequate precaution and peripheral blood mononuclear cells (PBMC) were separated from plasma by Ficoll-hypaque density gradient centrifugation, after washing, PBMC were cultured in tissue-culture medium without any stimulation for 48 h. Cell culture supernantant was collected and BCG-specific IgG antibodies were measured by ELISA. ALS positive [optical density (OD) ≥ 0.35] and ALS borderline positive (OD = 0.34) were categorized according to ALS cut-off for OD following basic principle described elsewhere (10).

### Measurements

Case report forms (CRF) were developed for collection of relevant data, and finalized after pre-testing. Characteristics analyzed include ALS, WHO criteria, and MKJC score.

### Analysis

All data were entered into a computer using SPSS for Windows (version 15.0; SPSS Inc., Chicago) and Epi-Info (version 6.0, USD, Stone Mountain, GA). Sensitivity, specificity, positive, and negative predictive values, and accuracy with their 95% confidence intervals (CIs) for the MKJC score, WHO criteria, and ALS were calculated by comparing with culture and/or Xpert MTB/RIF confirmed TB.

## Results

Among the 405 children enrolled during the study period, we analyzed MKJC score and WHO criteria for 388 children, ALS from blood sample for the first 221 children. Gastric lavage fluid and induced sputum was obtained from for microscopy (AFB), and mycobacterial culture for 396 children; real-time PCR by Xpert MTB/RIF for the last 214 children. The median (inter quartile) age of the study children was 10 (5, 16) months. In total of 27 (6.8%) children had microbiologically confirmed TB–in 10 by culture, in 21 by X-pert MTB/RIF, and in 4 by both the tests.

ALS was positive in 89/221 (40%) children (Table 1). Among 221 children who had ALS done 12 children had microbiologically confirmed TB–in eight by culture, in five by X-pert MTB/RIF, and in one by both the tests. TB was diagnosed by MKJC in 18% (68/388) and by WHO criteria in 17% (65/388) with an overlap of 65 TB cases (Table 1). Among 12 cases of “Confirmed TB” for whom ALS was done, 6 (50%) had positive ALS, 3 (25%) had borderline positive ALS, and 3 (25%) were negative for ALS. Among the children those who had “Not TB,” ALS was positive in 64 (38%), negative in 66 (39%), and borderline positive in 38 (23%) children. ALS was also positive in 19 cases that were diagnosed following clinical criteria (both by MKJC score and WHO criteria). Compared to culture and Xpert MTB/RIF confirmed TB, the sensitivity, specificity, positive predictive value, negative predictive value, and accuracy of MKJC score, WHO criteria as well as the ALS have been shown in Table 2.

**Table 1:** Summary results of modified Kenneth Jones criteria scoring, World Health Organization criteria and antibodies in lymphocyte supernatant in severely malnourished children under 5 years of age with TB.

**Parameters**	**Number of samples (one sample per patient)**	**Number of reports available**	**Positive for TB (%)**
ALS	221	221	89 (40)
MKJC score	388	388	68 (18)
WHO criteria	388	388	65 (17)

*ALS, Antibodies in Lymphocyte Supernatant; MKJC, modified Kenneth Jones criteria; WHO, World Health Organization*.

**Table 2:** Comparative performance of modified Kenneth Jones criteria scoring, World Health Organization criteria, and antibodies in lymphocyte supernatant for the diagnosing tuberculosis in severely malnourished children presenting with pneumonia.

**Parameter**	**Compared to culture positive MTB**					**Compared to culture and/or Gene X-pert positive TB**				
	**Sensitivity (95% CI) (%)**	**Specificity (95% CI) (%)**	**PPV (95% CI) (%)**	**NPV (95% CI) (%)**	**Accuracy**	**Sensitivity (95% CI) (%)**	**Specificity (95% CI) (%)**	**PPV (95% CI) (%)**	**NPV (95% CI) (%)**	**Accuracy**
ALS positive	50 (14–86)	60 (53–67)	3 (1–10)	98 (93–99)	60%	50 (14–86)	60 (53–67)	7 (3–14)	96 (90–98)	60%
MKJC positive	60 (27–86)	84 (79–87)	9 (4–19)	99 (97–100)	83%	37 (20–58)	84 (79–87)	15 (8–26)	95 (91–97)	79%
WHO positive	40 (14–73)	84 (80–87)	6 (2–16)	98 (96–99)	83%	22 (9–43)	83 (79–87)	9 (4–20)	93 (90–96)	79%

*MTB, Mycobacterium Tuberculosis; CI, Confidence Interval; ALS, Antibodies in Lymphocyte Supernatant; MKJC, modified Kenneth Jones criteria; WHO, World Health Organization; PPV, Positive Predictive Value; NPV, Negative Predictive Value*.

## Discussion

The most important observation of this study is the relative better specificity and accuracy of MKJC scoring and the WHO criteria in the diagnosis of TB in severely malnourished children with severe pneumonia. MKJC and WHO criteria are mainly based on clinical features, although chest radiography and sputum microscopy helps to improve the performance (5). The sensitivity of MKJC score and the WHO criteria ranged from 37 to 60% suggests that 40–63% of the TB cases will be missed if these criteria are used in the diagnosis in such study population. The specificity of around 84% suggests that only around 16% of the non-TB children would be inappropriately treated using them. It is important to note that TST is the cornerstone of both of the criteria and in this population false negativity of TST is high (12, 13), and, thus, once MKJC scoring or the WHO criteria is positive in this population the reliability of MKJC scoring and the WHO criteria is high. The results of the study also justify the use of MKJC scoring and the WHO criteria for the diagnosis of TB in severely malnourished children at the Dhaka Hospital of icddr,b since 1994 and 2006, respectively. Recently Bangladesh has adopted the WHO criteria in the national guideline for the management of TB in children (14). In our population, smear microscopy, one of the components of MKJC scoring and WHO criteria in diagnosing pulmonary tuberculosis, was often negative (only two culture confirmed smear positive cases among 396 children evaluated). Thus, clinicians heavily depend on the combination of clinical features, TST and chest radiography in applying these two criteria for diagnosis TB in this population.

The lesser performance of ALS in relation to MKJC scoring and the WHO criteria for the diagnosis of TB in our study population is interesting but critically important for the clinician in resource poor settings. The published data from the same cohort already revealed that ALS was not sufficiently accurate to improve the diagnosis of TB in severely malnourished children (15). However, that article did not reveal the comparative performance of ALS with MKJC scoring and the WHO criteria for the diagnosis of TB in our study population. Now, further analysis disclosed that the performance of MKJC scoring and the WHO criteria mainly based on clinical criteria compared to ALS was relatively better in diagnosing TB on the basis of their specificity. Thus, our study findings suggest that clinicians in resource limited settings may apply one of these two criteria, as the ALS assay performed poorly for the diagnosis and initiation of treatment for TB in severely malnourished children, especially with pneumonia as co-morbidity.

We have a limitation of our study which is the small sample of “Confirmed TB” and lack of evaluation of all study children with Xpert MTB/RIF assay that may warrant further studies with larger sample size. However, as we have a good number of Clinical TB cases on the basis of WHO clinical criteria and modified Kenneth Jones criteria (MKJC) score, we had ample of opportunities to adequately validate our clinical diagnostics compared to ALS for the diagnosis of TB in severely malnourished children. It is true that in our previously published article we have shown that ALS was not sufficiently accurate to improve the diagnosis of TB in severely malnourished children, but the objective of that article compared to prevailing one is totally different. Moreover, none of the tables or information in this article is reproduced from our previously published article (15). In this article, we have underscored on the evaluation for the use of clinical scoring in diagnosing TB in severely malnourished children compared to those using ALS but this evaluation was not performed in our previously published article on ALS (15). Although, there is common belief that the use of clinical criteria in severely malnourished children may not work and needs to rely on laboratory investigations for the diagnosis, we found that the use of clinical scoring may be better compared to immunological laboratory test for the diagnosis of childhood TB. As a number of previously published articles on ALS showed that it was good enough for the diagnosis of TB in adult (8, 9) and children (10) and is still used for the diagnosis of TB in some developing countries including Bangladesh. In the main work done by Raqib et al. (10), the role of ALS in diagnosing TB in children without severe malnutrition was found to be concordance with clinical as well as confirmed TB. However, the performance of ALS is questioned in our study mainly due to 38% positivity of ALS in children with “Not TB.” This might be due to latent TB infection from potential sub-clinical exposure of TB in TB endemic countries including Bangladesh as it is found in Interferon-Gamma Release Assays which cannot differentiate latent TB from active TB disease (16). Again, many immunological assays do not perform accurately especially in severely malnourished children with other underlying infections and thus the ALS assay appears to be inappropriate for diagnosis of TB in this special population of severely malnourished pediatric patients with pneumonia comorbidity. Thus, we felt this article might provide evidence that immunological assays such as ALS might not be suitable and sufficiently accurate in resource poor high TB burden settings where the value of the use of clinical scoring for the diagnosis of TB is still important.

In conclusion, despite low sensitivity of modified Kenneth Jones criteria score and WHO criteria for the diagnosis of TB in severely malnourished children, high specificity and accuracy of these two criteria, in relation to ALS, will make them useful for early diagnosis, prompt management, and prevention of complications, and saving money especially in resource limited TB endemic countries.

## Ethics Statement

This study (protocol number: PR-10067) was approved by the Research Review Committee (RRC) and the Ethical Review Committee (ERC) of icddr,b. Written informed consent was obtained from parents or guardians of each of the participating children; children whose caregivers did not give consent were not enrolled.

## Author Contributions

MC, AS, KS, SB, RR, LS, SI, S, HS, TA, MR, SN, FA, MS, and TA contributed in conceptualizing, design, data collection, data analysis, data interpretation, and manuscript writing. Additionally MC was responsible for attaining IRB approval and writing of the first draft of the manuscript. TA was responsible for overall supervision.

### Conflict of Interest

The authors declare that the research was conducted in the absence of any commercial or financial relationships that could be construed as a potential conflict of interest.
